# The association between disease activity and patient-reported outcomes in patients with moderate-to-severe ulcerative colitis in the United States and Europe

**DOI:** 10.1186/s12876-020-1164-0

**Published:** 2020-01-21

**Authors:** Alessandro Armuzzi, Miriam Tarallo, James Lucas, Daniel Bluff, Benjamin Hoskin, Danielle Bargo, Joseph C. Cappelleri, Leonardo Salese, Marco daCosta DiBonaventura

**Affiliations:** 1 Fondazione Policlinico A. Gemelli IRCCS – Università Cattolica del Sacro Cuore, Rome, Italy; 2 Pfizer Inc, Rome, Italy; 3 Adelphi Real World, Macclesfield, UK; 4 Pfizer Inc, New York, NY USA; 5 Pfizer Inc, Groton, CT USA; 6 Pfizer Inc, Collegeville, PA USA

**Keywords:** Ulcerative colitis, Quality of life, Work impairment, Activity impairment, Patient-reported outcomes

## Abstract

**Background:**

Patients with ulcerative colitis (UC) experience periods of recurring and episodic clinical signs and symptoms. This study sought to establish the association between disease activity and health-related quality of life (HRQoL) and other patient-reported outcomes.

**Methods:**

United States (US) and European Union 5 ([EU5]; i.e., France, Germany, Italy, Spain, and the United Kingdom) data from the 2015 and 2017 Adelphi Inflammatory Bowel Disease-Specific Programme (IBD-DSP) were used. The IBD-DSP is a database of retrospective patient chart information integrated with patient survey data (EuroQoL-5 Dimensions [EQ-5D], Short Quality of Life in Inflammatory Bowel Disease Questionnaire [SIBDQ], and Work Productivity and Activity Impairment-Ulcerative Colitis [WPAI-UC] questionnaire).

Using available chart information, physicians classified their moderate-to-severe patients into one of the following categories: remission with a Mayo endoscopic score = 0 (“deep remission”), remission without a Mayo endoscopic score = 0 (“remission”), or active disease. Differences among disease activity categories with respect to patient-reported outcomes were analyzed using generalized linear models, controlling for confounding variables.

**Results:**

*N* = 289 and *N* = 1037 patient charts with linked surveys were included from the US and EU5, respectively. The disease activity distribution was as follows: active disease = 40.1% (US) and 33.6% (EU5); remission = 48.0 and 53.0%; deep remission = 11.9 and 13.3%. Patients with active disease reported significantly lower levels of EQ-5D health state utilities (adjusted mean [AdjM] = 0.87 [US] and 0.78 [EU5]) compared with remission (AdjM = 0.92 and 0.91) and deep remission (AdjM = 0.93 and 0.91) (all *P* < 0.05 compared with active disease within each region). Similar findings were observed with the scores from the SIBDQ and the WPAI-UC. No significant differences were observed between remission categories.

**Conclusions:**

Among patients with moderate-to-severe UC in the US and EU5, active disease was associated with significant impairments in HRQoL, work and leisure activities. These results reinforce the importance, to both the patient and society, of achieving some level of remission to restore generic and disease-related HRQoL and one’s ability to work productively.

## Background

Affecting more than 900,000 people in the United States (US) and over 1.5 million people in Europe, ulcerative colitis (UC) is a chronic and debilitating inflammatory disease of the colon [1, 2]. Recent systematic reviews have suggested the incidence and prevalence of UC is globally on the rise [3]. The hallmark symptom of UC is urgency and bloody diarrhea, though patients often experience a number of other symptoms which can be either bowel-related (e.g., tenesmus, abdominal pain) or systemic (e.g., fatigue) [4, 5]. UC is also characterized by intermittent periods of disease flares and remission [4–6].

UC exerts a considerable burden on the patient and society. Those with UC report significantly poorer physical and mental health compared with general population samples [7]. Similarly, among patients with UC who are currently employed, an average of 31% of their work time is missed or rendered ineffective due to their health [7]. A separate database analysis in the US found indirect costs due to lost wages averaged between $4000 and $6000 per patient with UC per year, depending upon disease severity [8].

It is important to understand how the burden of UC may vary as a function of disease presentation. For example, several studies have explored the relationship between disease activity and patient-reported outcomes. A recent systematic review found that patients with active disease reported generic health status scores which were meaningfully lower than population norms, whereas no clinically meaningful burden was observed for patients in remission or post-surgery [9]. Similarly, observational studies in Europe have found significant associations between disease activity (measured by either the Simple Clinical Colitis Activity Index or the partial Mayo score) and generic and disease-specific health-related quality of life (HRQoL) [10–12]. However, the relationship between disease activity and work-related outcomes is less understood. An observational study in the United Kingdom found significantly higher levels of work impairment for patients with active disease compared with patients in remission; however, this study did not account for potentially confounding variables (e.g., comorbidities) [12]. To our knowledge, no other study has explored the impact of UC disease activity on work and leisure-related outcomes.

The aim of the present study was to examine, among those patients with a history of moderate-to-severe UC, the relationship between disease activity and several patient-reported outcomes including generic and disease-related QoL, work impairment, and leisure activity impairment. We included both the US and the five major countries of Europe (France, Germany, Italy, Spain, and the United Kingdom; EU5). The overall goal was to understand how the burden of moderate-to-severe UC varies across those who have active disease relative to patients in some form of disease remission.

## Methods

### Data sources

The present study used data from the 2015 and 2017 US and EU5 Inflammatory Bowel Disease (IBD)-Disease Specific Programme (DSP). The DSP data comprise retrospective medical chart information abstracted by each patient’s physician along with linked patient survey responses. The methods of the DSP have been previously published [13], though are summarized briefly below.

To acquire these data for the IBD-DSP, gastroenterologists in the US and EU5 were recruited by phone to participate in the study. Potential physician respondents were identified from publicly available lists of healthcare professionals. Field-based interviews were then conducted to ensure eligibility. Eligibility criteria included the following: gastroenterologists had to be board-certified, had to have been a qualified physician for between four and 40 years, had to make treatment decisions for more than eight patients with Crohn’s disease and seven patients with UC per month, and had to be active in the treatment management of their patients. Eligible gastroenterologists who agreed to participate in the IBD-DSP were then asked to complete patient record forms for the next seven consecutive eligible patients with UC.

Patients were eligible if they were 18 years of age or older, had a diagnosis of UC, had received either a steroid, immunomodulator (IM), or biologic for their UC, had been considered moderate or severe at some point based on the physician’s evaluation, and had a Mayo score of > 4 at some point. The patient record form was completed using an electronic data collection platform and included questions on the patient’s demographics and clinical data. Associated patients were then invited (participation was optional) to privately complete a paper-based patient self-completion form (i.e., patient survey), which included questions on demographics, their current condition, and patient-reported outcomes.

Study materials were piloted prior to study implementation to ensure sufficient content validity [12]. The protocol and study materials were reviewed and approved by the Western Institutional Review Board (Puyallup, WA).

### Sample

From the IBD-DSP database, our present study only included those who had completed the patient survey and met the eligibility criteria.

### Measures

#### Disease activity

Using available chart information (including endoscopy results, if they were available), physicians classified their patients into one of the three following mutually exclusive categories: 1) “deep remission”, defined as symptomatic remission with a Mayo endoscopic score of 0, 2) “remission”, defined as symptomatic remission without a Mayo endoscopic score of 0 (either no endoscopic data or a score of > 0), or 3) “active disease”. This variable served as the primary predictor.

#### Patient demographics and general health history

Country, age, sex, years diagnosed, body mass index, smoking status, and diagnosed comorbidities (used to calculate a Charlson Comorbidity Index) were also available from the patient record form and were included as covariates.

#### HRQoL

Two measures were used to assess HRQoL. Generic HRQoL was assessed using the EuroQoL-5 Dimension 3-Level (EQ-5D-3 L) instrument [14]. The EQ-5D-3 L is used to generate a health utility score, which varies conceptually from 0 (a health state associated with death) to 1 (a health state associated with perfect health), though negative values are possible for health states that are considered worse than death. The EQ-5D-3 L also includes a separate visual analog scale (VAS) which varies from 0 = worst health you can imagine to 100 = best health you can imagine [14]. Disease-specific HRQoL was assessed using the Quality of Life in Inflammatory Bowel Disease Questionnaire (IBDQ). The total Short IBDQ (SIBDQ) score was used (range 1 to 7), with higher scores indicating better HRQoL [15].

#### Work and activity impairment

To assess both work impairment and leisure activity impairment, the Work Productivity and Activity Impairment-Ulcerative Colitis (WPAI-UC), specific to UC, was used [16]. The six-item WPAI-UC generates four metrics: absenteeism (the percentage of work time missed due to the patient’s UC), presenteeism (the percentage of work time that was impaired while at work due to the patient’s UC), overall work impairment (the combination of absenteeism and presenteeism), and activity impairment (the percentage of non-work activities that were impaired due to the patient’s UC) [16].

### Statistical analysis

Analyses were conducted separately by region (US and EU5). Within each region, patients were stratified by disease activity and these categories of patients were compared with respect to demographics, general health history, and disease history using chi-square and one-way analysis of variance tests for categorical and continuous variables, respectively [17]. Variables that differed significantly among groups were included as potential confounding variables in subsequent regression models, as described below.

Disease activity was then used as the primary predictor of each patient-reported outcome measure in a series of generalized linear models specifying the appropriate distribution (e.g., normal distribution and identity link function for EQ-5D VAS, EQ-5D health utilities, and SIBDQ; negative binomial distribution and log link function for WPAI-UC measures [though a zero-inflated negative binomial model for WPAI-UC absenteeism was required due to model convergence issues]) [18]. All models controlled for country (EU5 analyses only), age, sex, body mass index, smoking status, years diagnosed, and the Charlson Comorbidity Index. Adjusted means are reported, along with 95% confidence intervals and the statistical significance relative to the reference category (active disease).

Analyses were conducted using all available outcomes data, rather than imposing case - wise deletion. In other words, patients did not need to have complete data on all outcomes to be included in the analysis; each regression model was conducted separately using all available data on that particular outcome.

## Results

### Sample characteristics

In the US, *N* = 289 patient charts with linked surveys were included in the analyses. These patients were 51.2% male with a mean age of 42.9 years (standard deviation [SD] = 14.9). Across the EU5, *N* = 1037 patients were included in the analyses. The demographic characteristics were generally similar to that of the US (55.6% male and a mean age of 39.2 years [SD = 13.8]). The complete list of characteristics for each region is reported in Table 1.

**Table 1:** Demographics of the study sample

	US	EU5
Country, n (%)		
US	289 (100.0)	–
EU5		1037 (100.0)
France	–	347 (33.5)
Germany	–	379 (36.5)
Italy	–	55 (5.3)
Spain	–	171 (16.5)
UK	–	85 (8.2)
Age, years		
Mean (SD)	42.9 (14.9)	39.2 (13.8)
Sex, n (%)		
Male	148 (51.2)	577 (55.6)
Female	141 (48.8)	460 (44.4)
Duration since diagnosis, years		
Mean (SD)	6.4 (7.0)	5.3 (5.9)
Body mass index		
Mean (SD)	25.7 (4.7)	24.0 (4.2)
Smoking status, n (%)		
Current smoker	23 (8.3)	151 (16.0)
Ex-smoker	88 (31.7)	295 (31.2)
Never smoked	167 (60.1)	499 (52.8)
Comorbidities, n (%)^a^		
Hypertension	73 (25.3)	111 (10.7)
Anxiety	54 (18.7)	110 (10.6)
Hyperlipidemia	46 (15.9)	69 (6.7)
Depression	33 (11.4)	55 (5.3)
Diabetes (with or without chronic complications)	21 (7.3)	30 (2.9)
Osteoarthritis	15 (5.2)	25 (2.4)
Anemia	10 (3.5)	61 (5.9)
Osteoporosis	9 (3.1)	24 (2.3)
Charlson Comorbidity Index score		
Mean (SD)	0.2 (0.6)	0.2 (0.6)

*Abbreviations: DSP* Disease Specific Programme, *EU5* European Union 5 (France, Germany, Italy, Spain, the UK), *SD* standard deviation, *UK* United Kingdom, *US* United States

^a^Only comorbidities that appear in > 2% of patients in either the US or EU5 are reported. Note that because multiple years of the DSP database were integrated into this analysis, comorbidities were not always assessed consistently in all patients. The percentages reflect the number of patients with a given comorbidity divided by the total number of patients who were assessed for that comorbidity (which may be lower than the total sample size in some cases)

**Fig. 1:**
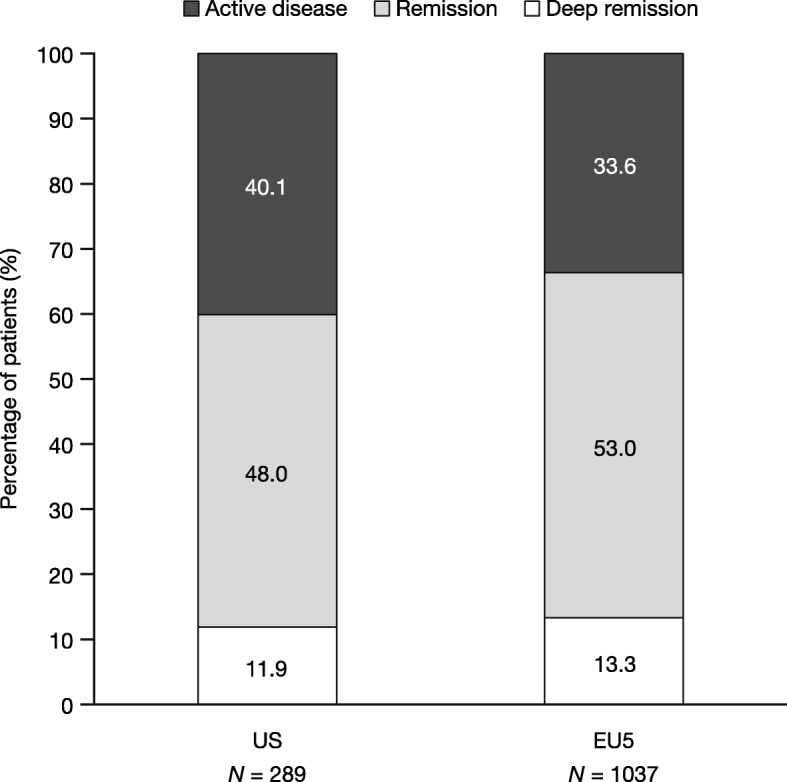
Distribution of remission status in the US and EU5. *Abbreviations: EU5* European Union 5 (France, Germany, Italy, Spain, the UK), *US* United States

Across both regions, only a minority of patients (11.9 and 13.3% in the US and EU5, respectively) were classified as being in “deep remission” based on their physician’s assessment. Approximately half (48.0 and 53.0%, respectively) were in remission with the remaining 40.1% in the US and 33.6% in the EU5 having active disease (Fig. 1).

### Disease activity and HRQoL

**Fig. 2:**
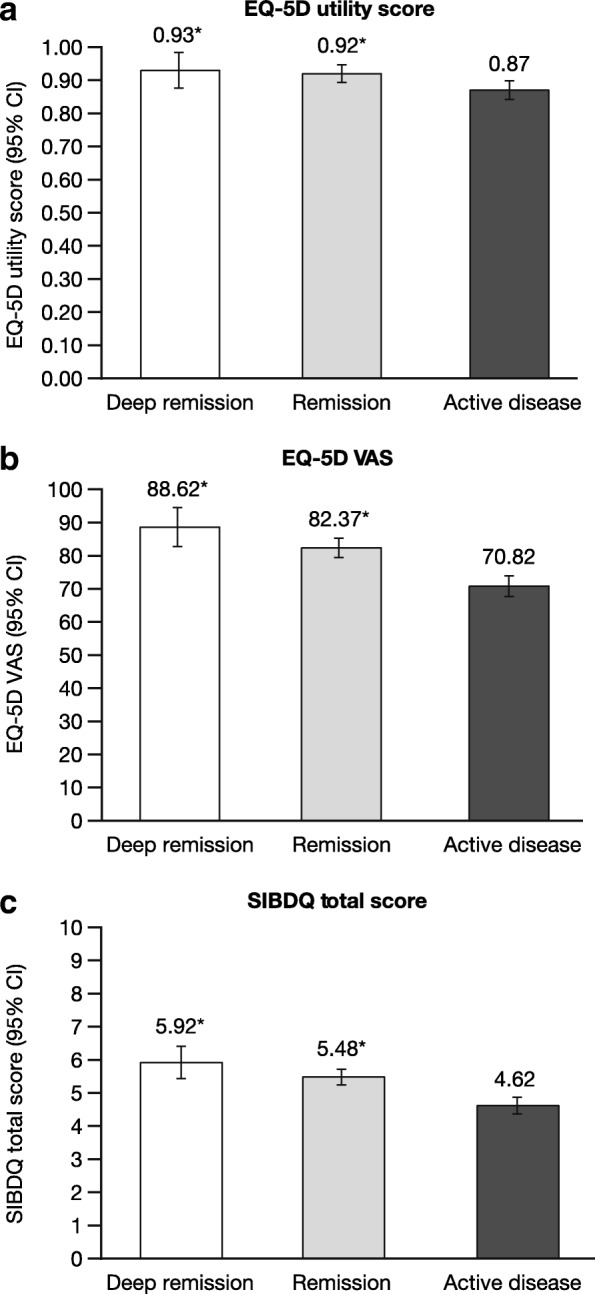
Overall and IBD-specific health-related quality of life by remission status in the US. **a.** EQ-5D utility score, **b.** EQ-5D VAS, and **c.** SIBDQ total score. *P < 0.05 relative to patients with active disease; all models controlled for age, sex, body mass index, smoking status, years diagnosed, and Charlson Comorbidity Index. *Abbreviations: CI* confidence interval, *EQ-5D* EuroQoL-5 Dimensions, *IBD* inflammatory bowel disease, *SIBDQ* Short Quality of Life in Inflammatory Bowel Disease Questionnaire, *US* United States, *VAS* visual analog scale

Significant associations between disease activity and the EQ-5D and SIBDQ were observed, even after adjusting for confounding variables (Table 2). In the US, patients with active disease reported significantly lower EQ-5D utility scores (adjusted mean = 0.87) compared with patients in remission (adjusted mean = 0.92) and deep remission (adjusted mean = 0.93) (both *P* < 0.05), though no differences were observed between remission and deep remission categories (Fig. 2). Similarly, patients with active disease also reported lower EQ-5D VAS and SIBDQ scores (adjusted means = 70.8 and 4.6, respectively) compared with patients in remission (adjusted means = 82.4 and 5.5) and deep remission (adjusted means = 88.6 and 5.9) (all *P* < 0.05). Again, no differences were observed between remission categories.

**Table 2:** Regression results predicting HRQoL in the US (a) and EU5 (b)

**a)**									
	**US**								
	**EQ-5D utility score** ***N* = 236**			**EQ-5D VAS** ***N* = 235**			**SIBDQ** ***N* = 232**		
	**b**	**95% CI**	***P***	**b**	**95% CI**	***P***	**b**	**95% CI**	***P***
Intercept	0.830	0.703, 0.958	< 0.001	74.730	60.705, 88.755	< 0.001	3.851	2.71, 4.993	< 0.001
Age	0.000	−0.002, 0.001	0.782	− 0.113	− 0.281, 0.056	0.189	0.006	− 0.008, 0.019	0.426
Female	−0.009	− 0.046, 0.028	0.641	−2.278	−6.384, 1.828	0.277	−0.071	− 0.408, 0.265	0.678
BMI	−0.001	−0.005, 0.003	0.664	−0.160	− 0.594, 0.273	0.469	0.009	−0.027, 0.044	0.626
Smoking status									
Current smoker^a^	–	–	–	–	–	–	–	–	–
Former smoker	0.096	0.025, 0.167	0.008	7.259	−0.552, 15.07	0.069	0.290	−0.345, 0.925	0.370
Never smoker	0.080	0.011, 0.148	0.023	8.480	0.929, 16.032	0.028	0.259	−0.354, 0.873	0.408
CCI	−0.045	− 0.082, − 0.007	0.020	−5.415	−9.399, −1.431	0.008	− 0.293	− 0.618, 0.032	0.077
Years diagnosed	0.000	−0.003, 0.004	0.759	−0.031	− 0.368, 0.306	0.856	0.023	−0.004, 0.051	0.096
Disease activity									
Active disease^a^	–	–	–	–	–	–	–	–	–
Remission	0.053	0.014, 0.093	0.008	11.554	7.191, 15.917	< 0.001	0.856	0.501, 1.211	< 0.001
Deep remission	0.065	0.004, 0.126	0.036	17.803	11.095, 24.511	< 0.001	1.299	0.746, 1.851	< 0.001
**b)**									
	**EU5**								
	**EQ-5D utility score** ***N* = 790**			**EQ-5D VAS** ***N* = 786**			**SIBDQ** ***N* = 758**		
	**b**	**95% CI**	***P***	**b**	**95% CI**	***P***	**b**	**95% CI**	***P***
Intercept	0.853	0.78, 0.926	< 0.001	69.701	61.817, 77.586	< 0.001	4.532	4.014, 5.05	< 0.001
Country									
France^a^	–	–	–	–	–	–	–	–	–
Germany	0.055	0.029, 0.081	< 0.001	1.500	−1.3, 4.299	0.294	0.067	−0.118, 0.252	0.477
Italy	0.069	0.016, 0.122	0.011	3.745	−1.932, 9.422	0.196	0.298	−0.084, 0.680	0.126
Spain	0.042	0.011, 0.073	0.009	0.230	−3.112, 3.572	0.893	−0.062	−0.281, 0.157	0.578
UK	0.011	−0.036, 0.058	0.636	−0.617	−5.606, 4.372	0.808	−0.337	−0.659, − 0.016	0.040
Age	−0.001	−0.002, 0.000	0.050	−0.066	− 0.164, 0.032	0.185	− 0.007	−0.013, 0.000	0.051
Female	−0.027	−0.049, − 0.006	0.014	−1.582	−3.934, 0.770	0.187	− 0.215	−0.37, − 0.06	0.007
BMI	−0.002	−0.004, 0.001	0.166	−0.075	− 0.352, 0.201	0.593	0.001	−0.017, 0.019	0.914
Smoking status									
Current smoker^a^	–	–	–	–	–	–	–	–	–
Former smoker	− 0.019	− 0.051, 0.013	0.247	−3.896	−7.341, − 0.450	0.027	− 0.175	− 0.405, 0.054	0.135
Never smoker	0.001	−0.029, 0.031	0.942	−0.809	−4.005, 2.387	0.620	0.072	−0.142, 0.286	0.509
CCI	−0.004	−0.023, 0.015	0.673	−2.451	−4.572, − 0.331	0.023	− 0.143	−0.286, 0.001	0.051
Years diagnosed	−0.002	− 0.004, 0.000	0.122	− 0.211	− 0.424, 0.001	0.051	0.002	− 0.012, 0.016	0.784
Disease activity									
Active disease^a^	–	–	–	–	–	–	–	–	–
Remission	0.131	0.108, 0.154	< 0.001	17.034	14.529, 19.538	< 0.001	1.386	1.221, 1.552	< 0.001
Deep remission	0.127	0.094, 0.161	< 0.001	18.191	14.596, 21.785	< 0.001	1.558	1.322, 1.795	< 0.001

*Abbreviations: b* regression estimate, *BMI* body mass index, *CCI* Charlson Comorbidity Index, *CI* confidence interval, *EQ-5D* EuroQoL-5 Dimensions, *EU5* European Union 5 (France, Germany, Italy, Spain, the UK), *HRQoL* health-related quality of life, *SIBDQ* Short Quality of Life in Inflammatory Bowel Disease Questionnaire, *UK* United Kingdom, *US* United States, *VAS* visual analog scale

^a^Indicates reference category

**Fig. 3:**
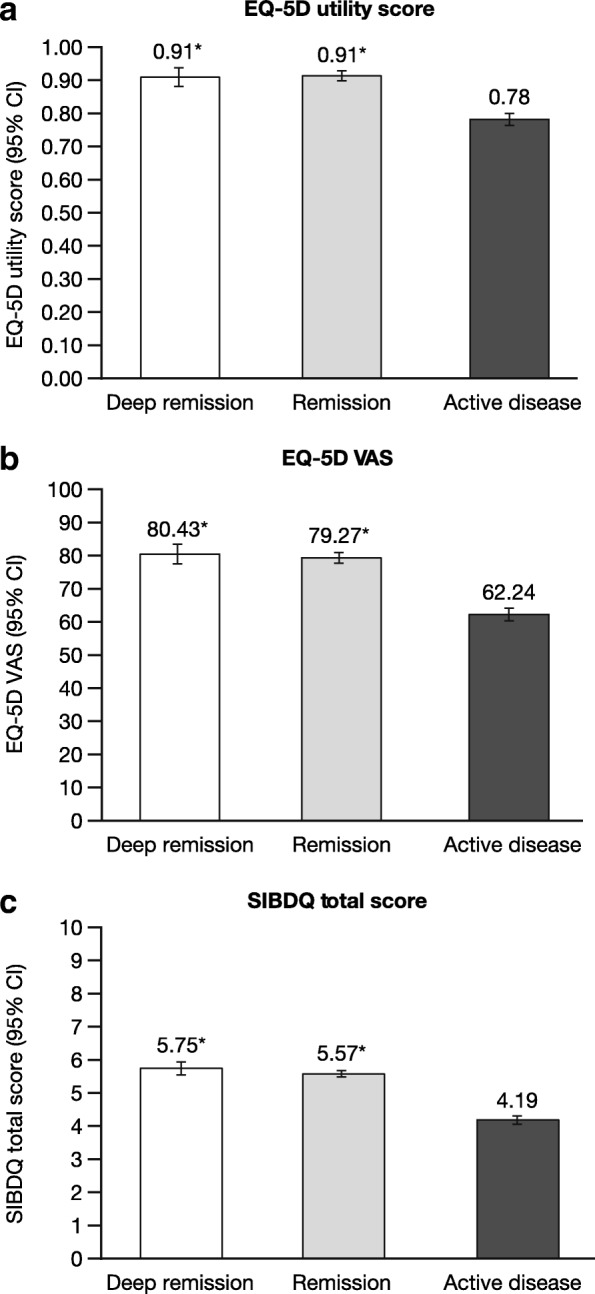
Generic and disease-specific health-related quality of life by remission status in the EU5. Values presented are adjusted means. **a.** EQ-5D utility score, **b.** EQ-5D VAS, and **c.** SIBDQ total score. *P < 0.05 relative to patients with active disease; all models controlled for country, age, sex, body mass index, smoking status, years diagnosed, and Charlson Comorbidity Index. *Abbreviations: CI* confidence interval, *EQ-5D* EuroQoL-5 Dimensions, *EU5* European Union 5 (France, Germany, Italy, Spain, the UK), *SIBDQ* Short Quality of Life in Inflammatory Bowel Disease Questionnaire, *VAS* visual analogue scale

The same pattern was observed among patients in the EU5 (Fig. 3). Patients with active disease reported significantly lower EQ-5D utility and EQ-5D VAS scores (adjusted means = 0.78 and 62.2, respectively) compared with patients in remission (adjusted mean = 0.91 and 79.3) and deep remission (adjusted mean = 0.91 and 80.4) (all *P* < 0.05). Patients with active disease in the EU5 also reported lower scores of the SIBDQ (adjusted mean = 4.2) compared with both remission (adjusted mean = 5.6) and deep remission (adjusted mean = 5.7) categories (both *P* < 0.05).

### Disease activity and work and activity impairment

**Fig. 4:**
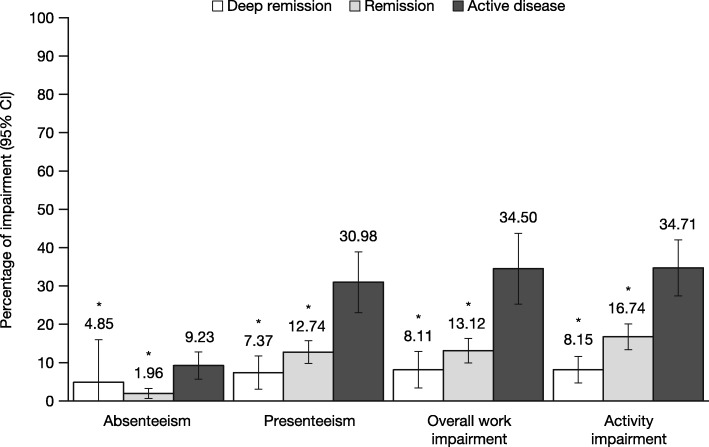
Adjusted levels of work- and activity-related impairment by remission status in the US. *P < 0.05 relative to patients with active disease; all models controlled for age, sex, body mass index, smoking status, years diagnosed, and Charlson Comorbidity Index. *Abbreviations: CI* confidence interval, *US* United States

**Fig. 5:**
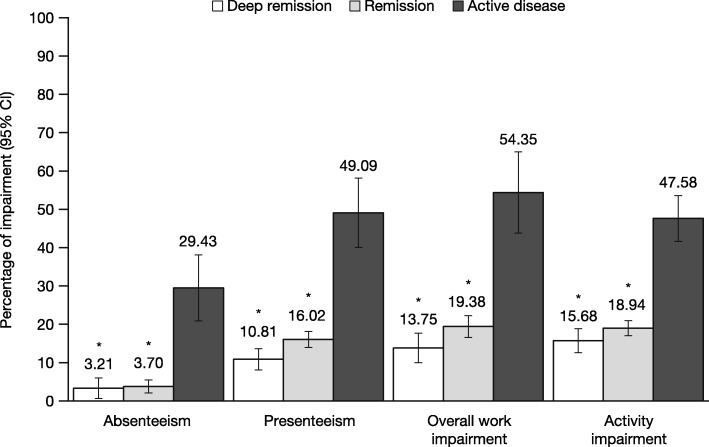
Adjusted levels of work and activity-related impairment by remission status in the EU5. *P < 0.05 relative to patients with active disease; all models controlled for country, age, sex, body mass index, smoking status, years diagnosed, and Charlson Comorbidity Index. *Abbreviations: CI* confidence interval, *EU5* European Union 5 (France, Germany, Italy, Spain, the UK)

Across both regions, significant associations between disease activity and work-related outcomes were observed, even after adjusting for confounding variables (Table 3). As with HRQoL, the poorest outcomes were observed among those with active disease. Patients with active disease in the US and EU5 reported the highest levels of presenteeism (adjusted means = 31.0 and 49.1%, respectively), overall work impairment (adjusted means = 34.5 and 54.4%), and activity impairment (adjusted means = 34.7 and 47.6%) (Figs. 4 and 5). Patients with active disease in the US had significantly higher levels of absenteeism than patients in remission (adjusted means = 9.2% vs. 2.0%, *P* < 0.05), but no difference was observed between patients with active disease and those in deep remission (though patients with active disease had numerically higher levels of absenteeism) (Fig. 4). Patients with active disease in the EU5 reported the highest levels of absenteeism (adjusted mean = 29.4%), which was significantly greater than both remission groups (Fig. 5).

**Table 3:** Regression results predicting work and activity impairment in the US (a) and EU5 (b)

**a)**												
	**US**											
	**Absenteeism** ***N* = 156**			**Presenteeism** ***N* = 162**			**Overall work impairment** ***N* = 152**			**Activity impairment** ***N* = 232**		
	**b**	**95% CI**	***P***	**b**	**95% CI**	***P***	**b**	**95% CI**	***P***	**b**	**95% CI**	***P***
Intercept	4.308	2.693, 5.923	< 0.001	3.679	2.182, 5.177	< 0.001	4.077	2.573, 5.580	< 0.001	3.612	2.722, 4.502	< 0.001
Age	−0.002	−0.031, 0.026	0.864	− 0.004	− 0.020, 0.012	0.617	− 0.002	− 0.019, 0.014	0.782	− 0.002	− 0.014, 0.010	0.770
Female	0.587	0.111, 1.063	0.016	−0.047	− 0.399, 0.305	0.794	−0.090	− 0.450, 0.270	0.624	0.097	−0.180, 0.374	0.492
BMI	−0.019	−0.089, 0.051	0.595	0.007	−0.042, 0.055	0.790	0.001	− 0.047, 0.048	0.983	0.008	−0.020, 0.037	0.568
Smoking status												
Current smoker^a^	–	–	–	–	–	–	–	–	–	–	–	–
Former smoker	− 0.460	−1.214, 0.294	0.231	0.071	− 0.610, 0.752	0.838	− 0.130	− 0.870, 0.609	0.730	− 0.179	− 0.712, 0.354	0.511
Never smoker	−0.401	−1.115, 0.313	0.271	−0.156	− 0.826, 0.513	0.648	− 0.348	− 1.074, 0.378	0.348	− 0.164	− 0.666, 0.338	0.522
CCI	− 0.137	− 0.636, 0.361	0.589	0.014	−0.368, 0.396	0.942	0.053	−0.335, 0.440	0.790	0.216	−0.028, 0.460	0.083
Years diagnosed	−0.139	− 0.203, − 0.075	< 0.001	−0.030	− 0.064, 0.004	0.086	− 0.033	−0.068, 0.002	0.066	−0.024	− 0.049, 0.001	0.057
Disease activity												
Active disease^a^	–	–	–	–	–	–	–	–	–	–	–	–
Remission	−0.906	−1.456, − 0.356	0.001	− 0.889	−1.247, − 0.531	< 0.001	−0.967	− 1.340, − 0.593	< 0.001	−0.730	−1.028, − 0.431	< 0.001
Deep remission	0.964	−0.408, 2.336	0.169	−1.436	−2.078, − 0.793	< 0.001	− 1.447	− 2.091, − 0.804	< 0.001	−1.449	− 1.922, − 0.977	< 0.001
**b)**												
	**EU5**											
	**Absenteeism** ***N* = 404**			**Presenteeism** ***N* = 452**			**Overall work impairment** ***N* = 379**			**Activity impairment** ***N* = 771**		
	**b**	**95% CI**	***P***	**b**	**95% CI**	***P***	**b**	**95% CI**	***P***	**b**	**95% CI**	***P***
Intercept	4.307	3.027, 5.586	< 0.001	3.838	3.099, 4.578	< 0.001	4.039	3.256, 4.822	0.000	3.904	3.404, 4.404	< 0.001
Country												
France^a^	–	–	–	–	–	–	–	–	–	–	–	–
Germany	−0.184	− 0.613, 0.245	0.400	− 0.472	− 0.708, − 0.236	< 0.001	−0.283	− 0.542, − 0.025	0.031	−0.297	− 0.479, − 0.116	0.001
Italy	− 1.415	−2.236, − 0.594	0.001	− 0.276	−0.887, 0.334	0.375	−0.040	− 0.665, 0.585	0.900	− 0.302	− 0.662, 0.059	0.101
Spain	−0.638	− 1.116, − 0.160	0.009	− 0.054	−0.360, 0.253	0.732	0.083	−0.239, 0.406	0.613	−0.137	−0.350, 0.076	0.208
UK	−0.366	−0.972, 0.240	0.236	−0.435	− 0.884, 0.014	0.058	− 0.328	−0.794, 0.139	0.169	0.104	−0.213, 0.421	0.520
Age	0.004	−0.013, 0.021	0.649	0.007	−0.004, 0.019	0.191	0.005	−0.007, 0.016	0.416	0.006	−0.001, 0.012	0.074
Female	−0.053	−0.395, 0.289	0.762	0.105	−0.100, 0.310	0.317	0.097	−0.129, 0.323	0.400	0.052	−0.102, 0.205	0.511
BMI	0.006	−0.037, 0.050	0.777	0.003	−0.021, 0.028	0.789	0.002	−0.024, 0.027	0.896	−0.004	−0.022, 0.013	0.631
Smoking status												
Current smoker^a^	–	–	–	–	–	–	–	–	–	–	–	–
Former smoker	−0.055	−0.523, 0.414	0.819	0.203	−0.077, 0.483	0.155	0.240	−0.071, 0.551	0.130	0.207	−0.016, 0.430	0.069
Never smoker	−0.303	−0.760, 0.155	0.195	−0.045	− 0.310, 0.219	0.737	− 0.009	−0.296, 0.277	0.949	−0.043	− 0.251, 0.166	0.688
CCI	0.392	−0.028, 0.812	0.067	−0.074	−0.294, 0.145	0.506	−0.046	− 0.289, 0.197	0.708	0.163	0.027, 0.299	0.019
Years diagnosed	−0.036	−0.073, 0.002	0.061	−0.034	− 0.052, − 0.015	< 0.001	−0.055	− 0.077, − 0.033	< 0.001	−0.027	− 0.040, − 0.013	< 0.001
Disease activity												
Active disease^a^	–	–	–	–	–	–	–	–	–	–	–	–
Remission	−0.982	−1.371, − 0.592	< 0.001	−1.120	−1.342, − 0.899	< 0.001	−1.031	− 1.272, − 0.79	< 0.001	−0.921	− 1.082, − 0.761	< 0.001
Deep remission	−1.063	− 1.681, − 0.445	0.001	−1.513	− 1.837, − 1.189	< 0.001	−1.374	− 1.722, − 1.027	< 0.001	−1.110	− 1.348, − 0.872	< 0.001

*Abbreviations: b* regression estimate, *BMI* body mass index, *CCI* Charlson Comorbidity Index, *CI* confidence interval, *EU5* European Union 5 (France, Germany, Italy, Spain, the UK), *UK* United Kingdom, *US* United States

^a^Indicates reference category

## Discussion

The objective of the current study was to examine the effect of disease activity on patient-reported outcomes in the US and EU5 among those patients with moderate-to-severe UC. A number of studies have documented the overall burden of UC with respect to HRQoL and the ability to work productively [7–12]; however, our study focused on the relationship between disease activity and these outcomes controlling for confounding variables.

More than a third of patients in our study were classified as having active disease by their gastroenterologist despite, by definition, having received a steroid or advanced therapy (either an IM or a biologic). These patients with active disease reported significantly lower levels of HRQoL, both generic (EQ-5D) and disease-specific (SIBDQ), and significantly higher levels of work and leisure-related impairment (WPAI-UC). Although a formal evaluation of indirect cost estimates is beyond the scope of this study, using a human capital approach, the levels of overall work impairment we observed would equate to $15,400 in lost wages per employed patient with UC per year in the US (based on annualized 2017 median weekly earnings from the US Bureau of Labor Statistics [19]) and €11,430 in lost wages per employed patient with UC per year in the EU5 (based on annualized 2016 hourly median earnings from Eurostat [20]).

The pattern of results was observed consistently across both regions. The size of the effects was noteworthy. The decrements observed in generic HRQoL among those with active disease surpassed cutoffs for what would be considered minimal clinically important differences for the EQ-5D [21]. Further, between a third and a half of work and leisure time of patients with active disease was rendered ineffective due to their UC, which was at least two to three times higher than what was observed for patients in either remission group.

It is worth noting that the mean values for the active disease group were often higher than what has been observed in prior research. For example, a Polish study by Kawalec et al. (2018) reported EQ-5D utility values for those in remission that were similar to the values for our study’s two remission groups; however, their utility values for patients with active disease were much lower than in our study (> 0.10 points lower) [22]. Similarly, a multi-centered European study by Van Assche et al. (2018) reported EQ-5D utility values for patients who perceived themselves to be “normal or in remission” that were similar to the values for our study’s two remission groups but, again, reported utility values for severe patients that were much lower than our study’s patients with active disease (> 0.10 points lower than our active disease group) [23]. In some cases, the same was true for SIBDQ scores and, to a lesser extent, WPAI-UC scores [23]. The reasons for the differences are unclear and warrant additional investigation. It is possible that our regression approach (e.g., controlling for a comorbidity index) partialed out more variance in these outcomes than other studies, thus diminishing the effect of disease activity on utility scores and lowering the differences in adjusted mean values.

It is also possible that the subjective interpretation of “active disease” might have been applied more broadly in our study. That is, physicians in our study may have been disproportionately more likely than physicians in other studies to classify less severe patients in the active disease group. More research is necessary though these comparisons suggest our study, if anything, may underestimate the burden of active disease on patient-reported outcomes.

Interestingly, few differences were observed between the two remission groups (deep remission vs. remission). The reasons may be purely methodological. For example, to be classified as being in “deep remission”, a patient needed to have an endoscopic score of 0. If a patient did not have such data available, then they could not be considered in “deep remission”. Therefore, patients with the same underlying level of disease activity may be categorized in either remission category based on the presence versus absence of supportive endoscopic data. Unfortunately, we do not know how frequently this occurred to further test this post-hoc hypothesis. It is also possible that patient-reported outcomes legitimately do not vary by remission category. There is some preliminary evidence of this in a study by Panés et al. (2017); levels of EQ-5D varied very little across low levels of patient and physician Simple Clinical Colitis Activity Index values despite an overall significant relationship between the variables [10]. This would suggest that, despite the incremental clinical benefit to achieving mucosal healing versus resolution of patient symptoms without mucosal healing, patient-reported outcomes may not fully reflect these distinctions. Further research is necessary.

### Limitations

Several limitations should be noted. Our measure of disease activity was intentionally subjective, to allow discretion on the part of the gastroenterologist to rely on the pieces of patient information most relevant. Although we would argue this may be ideal for real-world research endeavors (where collection of data can vary substantially across practices), it limits the interpretability of what aspects of disease activity are most predictive of patient-reported outcomes. It also potentially created bias as patients without endoscopy results could not be considered in “deep remission”, by definition, hence there is a risk of measurement error between the “remission” and “deep remission” groups. However, the results suggest that any state of remission is notably different to active disease. Additionally, data on the availability of endoscopy results was not specifically collected, and hence the proportion of patients with available endoscopy results is unknown. Furthermore, the approach used to analyze the outcome data by using all available outcome data rather than casewise deletion could have potentially created bias in the estimation of the effect of disease activity on patient-reported outcomes.

Our study did account for a variety of confounding variables, which would undoubtedly be associated with patient-reported outcomes; however, we could only rely on what was included in the DSP data. For example, treatment history (which exists in the database) was not included as a confounding variable as not all respondents had a complete treatment history available. Although all patients were invited to provide patient-reported outcomes, only a subsample of patients did so, and the extent to which this subpopulation is systematically different from the pool of patients who were given the opportunity remains unclear (though post-hoc analyses uncovered no differences with respect to demographic and clinical variables between those who did and did not complete other than a lower body mass index for those who completed the survey).

Finally, our study included data from both the US and EU5, although it was not designed to systematically assess differences in our research question across regions. It would appear there is a greater burden among patients in the EU5 as patients with active disease in the EU5 reported numerically lower levels of HRQoL and more work and leisure activity impairment than patients with active disease in the US. However, patients in the EU5 were younger, diagnosed for longer, and more likely to be male than patients in the US. There may be other unobserved differences which could explain the regional differences in patient outcomes. Further research would be necessary to explore these questions.

## Conclusions

Among patients with moderate to severe UC in the US and EU5, over a third of patients managed by gastroenterologists had active disease despite treatment with an IM or a biologic. Active disease was associated with significant impairments in HRQoL and in work and leisure activities. These results reinforce the importance, to both the patient and society, of achieving some level of remission to restore generic and disease-related QoL and one’s ability to work productively.

## Data Availability

In this study, data from the 2015 and 2017 Adelphi Inflammatory Bowel Disease Specific Programme (IBD-DSP) were used. Permissions were required and obtained from Adelphi to use the Adelphi IBD Disease Specific Programme dataset.

## Abbreviations

AdjM: Adjusted mean
CI: Confidence interval
DSP: Disease Specific Programme
EQ-5D: EuroQoL-5 Dimensions
EU5: European Union 5 (France, Germany, Italy, Spain, the UK)
HRQoL: Health-related quality of life
IBD: Inflammatory bowel disease
IBD-DSP: Inflammatory Bowel Disease Specific Programme
IM: Immunomodulator
SD: Standard deviation
SIBDQ: Short Quality of Life in Inflammatory Bowel Disease Questionnaire
UC: Ulcerative colitis
UK: United Kingdom
US: United States
VAS: Visual analog scale
WPAI-UC: Work Productivity and Activity Impairment-Ulcerative Colitis
